# Bioimpedance in CKD: an untapped resource?

**DOI:** 10.1093/ndt/gfac275

**Published:** 2022-10-19

**Authors:** Kaitlin J. Mayne, Jennifer S. Lees, William G. Herrington

**Affiliations:** 1 Medical Research Council Population Health Research Unit, Clinical Trial Service Unit and Epidemiological Studies Unit, Nuffield Department of Population Health, University of Oxford, Oxford, UK; 2 School of Cardiovascular and Metabolic Health, College of Medical and Veterinary Life Sciences, University of Glasgow, Glasgow, UK

## What is Bioimpedance Spectroscopy?

Bioimpedance devices estimate body water and fat by mesauring opposition to an electrical current applied via the skin. The most commonly reported device from existing dialysis literature is the Fresenius Body Composition Monitor (BCM), which uses bioimpedance spectroscopy (BIS). Although sometimes used interchangeably, BIS is not synonymous with bioimpedance analysis (BIA). BIA traditionally used only a single frequency before multifrequency BIA devices were developed, which measure impedance at 50–200 discrete frequencies from 3 to 1000 kHz. BIS extends this range by extrapolation to zero and infinity kHz. Greater frequency range improves discrimination of extracellular (ECW) from intracellular water. All BIS/BIA devices also estimate lean and fat tissue, but the BCM uniquely specifically quantifies fluid overload independent of body composition using the three-compartment model [1].

## Fluid Overload Parameters and Relevance to Mortality

The BCM reports two key parameters: absolute fluid overload in litres and percentage relative fluid overload (the absolute value as a proportion of ECW). These parameters are the most widely studied bioimpedance indices in kidney disease cohorts but are unique to the BCM device. Measurement in litres is arguably more meaningful to dialysis clinicians, while relative fluid overload may be favoured in research to simplify between-individual comparisons. Based on a healthy population, the normal limits of BCM-measured fluid status (i.e. euvolemia) are ±1.1 L [2]. In dialysis studies, a two-tier approach has emerged where >1.1–2.5 L (approximately equivalent to >7–15% relative fluid overload) is often considered moderate and >2.5 L (approximately >15%) is considered severe fluid overload. Compared with euvolemia, moderate pre-haemodialysis fluid overload has been associated with an ~60% increased risk of death {adjusted hazard ratio [HR] 1.64 [95% confidence interval (CI) 1.35–1.98]} [3], and in metaanalyses of other studies in both haemodialysis and peritoneal dialysis cohorts, severe fluid overload was associated with about a doubling of mortality [HR 2.28 (95% CI 1.56–3.34)] [4]. Such associations are unmodified at different levels of blood pressure [5], suggesting an independent role for fluid overload as a potential cause of mortality.

## Practical use of Bioimpedance in Kidney Failure with Replacement Therapy (KFRT)

Clinically, accurate volume assessment is an essential component of dialysis prescription. Although routine clinical assessments may often be sufficient to avoid extremes of hydration status, adjunctive BCM assessments have theoretical advantages in KFRT. Tracking fluid status using a BCM can be achieved with minimal training and should provide objective measures with less potential for between-observer differences than clinical assessments. When used in peritoneal dialysis, the presence or absence of indwelling dialysate is not clinically important if serial measurements follow a consistent approach.

Small randomised controlled trials have shown that using bioimpedance (both BIS and BIA) improves volume status and systolic blood pressure relative to usual care [6, 7]. However, trials have not found that bioimpedance assessments reduce the risk of intradialytic hypotension [7], nor do they preserve residual kidney function compared with routine care [8]. No trial has been sufficiently large to test the effects on the risk of hospitalisation or mortality-based outcomes.

In research, the BCM has been used to assess eligibility and outcomes. In the BVM-Reg trial of different techniques to monitor ultrafiltration (NCT01416753), BCM-assessed severe fluid overload ≥15% was an inclusion criterion. The SOLiD trial (ACTRN12611000975998) found that although allocation to a lower dialysate sodium of 135 mmol/L versus 140 mmol/L did not lead to any significant effect on the primary outcome of left ventricular mass index, the intervention did reduce BCM-measured ECW by ∼0.6 L over the 12-month trial.

## Bioimpedance in CKD without Kidney Replacement Therapy (KRT)

The use of bioimpedance is relatively unexplored in CKD without KRT, but observational studies are emerging. These have focused on moderate fluid overload (>1.1 L or >7%) as an exposure since severe fluid overload is relatively uncommon before kidney failure develops. In a recent systematic review we identified 11 CKD without KRT cohorts reporting associations between bioimpedance indices of fluid overload and mortality, cardiovascular outcomes and/or CKD progression [9]. There is consistent evidence of positive associations between moderate fluid overload with these adverse cardiorenal outcomes [9]. Formal observational meta-analysis was not possible due to substantial variation in bioimpedance methods and fluid overload definitions. Establishing a consensus approach using consistent terminology would facilitate research and clinical adoption.

Bioimpedance is also being explored in CKD research: the placebo-controlled EMPA-KIDNEY trial (NCT03594110; www.empakidney.org) is assessing whether empagliflozin rdeuces the risk of a composite of kidney disease progression or cardiovascular death in 6609 participants with CKD. In a substudy of ~ 10% of participants, BCM has been used to assess the effects of empagliflozin on fluid overload and adiposity. The substudy includes a secondary outcome combining clinical outcomes with bioimpedance data. The outcome is defined as a composite of death due to heart failure, hospitalisation for heart failure or the development of new moderate (>7%) or severe (>15%) fluid overload.

Another feature of the current published literature using bioimpedance in CKD without KRT is a lack of phenotyping for coexistent heart failure, with bioimpedance studies in heart failure cohorts also often overlooking the consideration of kidney parameters [9]. In early CKD, even 1 L excess ECW measured by BIS has been associated with the development of left ventricular hypertrophy and diastolic dysfunction [10]. Such levels of fluid overload may be difficult to detect clinically. Subclinical BCM-detected fluid overload could be used as a trigger for screening for heart failure and guide the use of diuretics.

Despite fluid overload being a hallmark of heart failure, bioimpedance is not widely used nor is it well tested in randomised trials. A small feasibility study of at-home BIA in heart failure is ongoing (NCT05177081), which may provide justification for the use of bioimpedance as a component of telemedicine consultations. Unique to the heart failure setting, intra- and transthoracic impedance are also available via some devices used for rhythm monitoring.

## Conclusions

**Figure 1:**
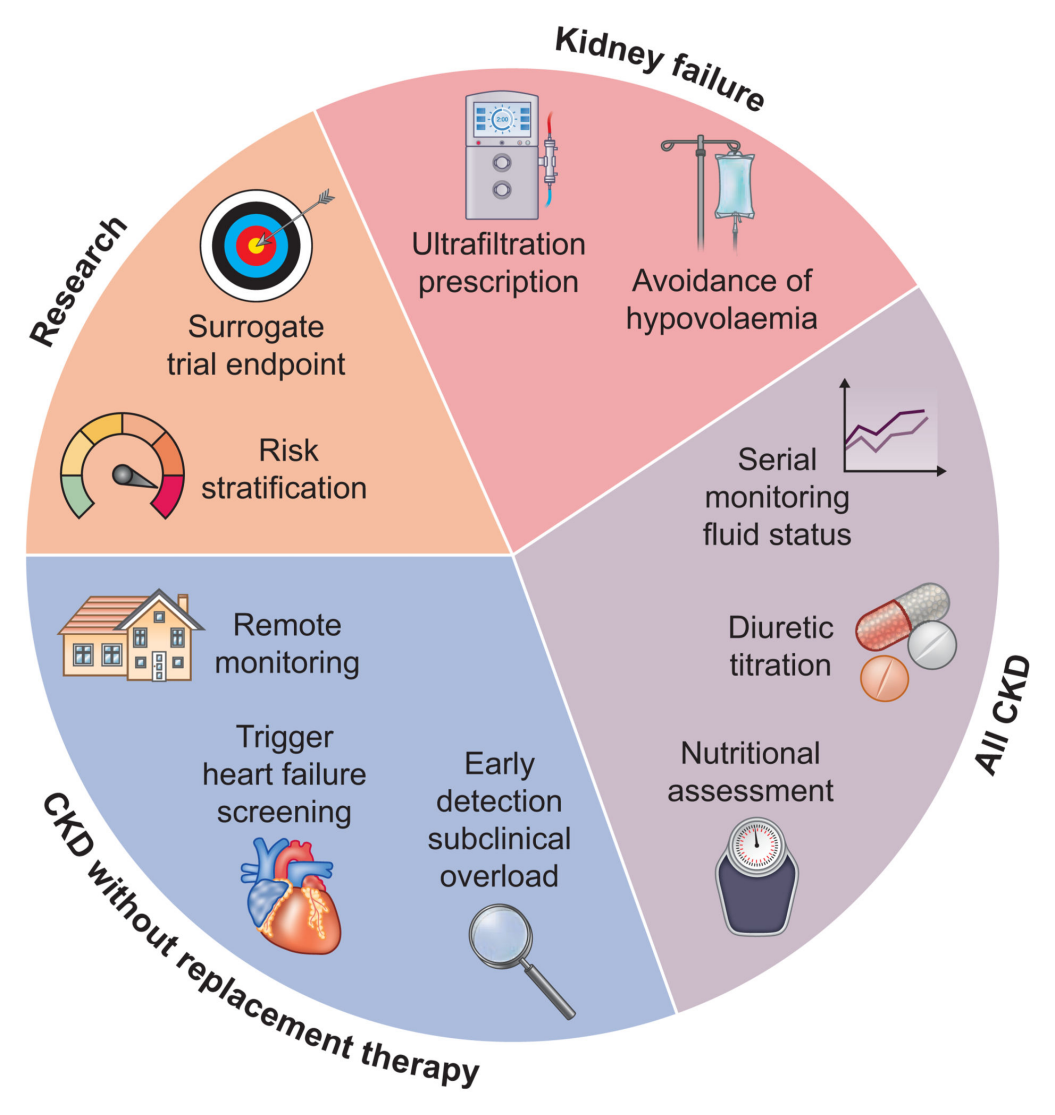
Potential roles for Bioimpedance Spectroscopy in CKD.

Bioimpedance devices—and particularly the BCM—have a range of potential clinical and research applications in a range of patient groups, not just kidney failure with replacement therapy (Fig. 1). We envisage particular value where CKD and heart failure coexist and encourage development of consensus on optimum methods and terminology to improve research and implementation.

## Data Availability

Data sharing is not applicable to this article as no datasets were generated or analysed during the current study.
